# Design of a Waterborne Polyurethane–Urea Ink for Direct Ink Writing 3D Printing

**DOI:** 10.3390/ma14123287

**Published:** 2021-06-14

**Authors:** Julen Vadillo, Izaskun Larraza, Tamara Calvo-Correas, Nagore Gabilondo, Christophe Derail, Arantxa Eceiza

**Affiliations:** 1 Materials + Technologies Research Group (GMT), Department of Chemical and Environmental Engineering, Faculty of Engineering of Gipuzkoa, University of Basque Country, Plz. Europa 1, 20018 Donostia-San Sebastian, Spain; julen.vadillo@univ-pau.fr (J.V.); izaskun.larraza@ehu.eus (I.L.); tamara.calvo@ehu.eus (T.C.-C.); nagore.gabilondo@ehu.eus (N.G.); 2 Universite de Pau et Pays de l’Adour, E2S UPPA, CNRS, IPREM, UMR5254, Institut des Sciences Analytiques & de PhysicoChimie pour l’Environnement & les Matériaux, 64000 Pau, France

**Keywords:** waterborne polyurethane–urea ink, 3D printing, shape fidelity, printability, rheology, solvent-free

## Abstract

In this work, polycaprolactone–polyethylene glycol (PCL–PEG) based waterborne polyurethane–urea (WBPUU) inks have been developed for an extrusion-based 3D printing technology. The WBPUU, synthesized from an optimized ratio of hydrophobic polycaprolactone diol and hydrophilic polyethylene glycol (0.2:0.8) in the soft segment, is able to form a physical gel at low solid contents. WBPUU inks with different solid contents have been synthesized. The rheology of the prepared systems was studied and the WBPUUs were subsequently used in the printing of different pieces to demonstrate the relationship between their rheological properties and their printing viability, establishing an optimal window of compositions for the developed WBPUU based inks. The results showed that the increase in solid content results in more structured inks, presenting a higher storage modulus as well as lower tan δ values, allowing for the improvement of the ink’s shape fidelity. However, an increase in solid content also leads to an increase in the yield point and viscosity, leading to printability limitations. From among all printable systems, the WBPUU with a solid content of 32 wt% is proposed to be the more suitable ink for a successful printing performance, presenting both adequate printability and good shape fidelity, which leads to the realization of a recognizable and accurate 3D construct and an understanding of its relationship with rheological parameters.

## 1. Introduction

Direct ink writing (DIW) 3D printing is an additive manufacturing technique that has generated increasing interest in many fields in the last year, owing to the possibility of avoiding the limitations of other 3D printing techniques such as the use of volatile organic compounds or the need for high temperatures for its processing; moreover, this technique offers high resolution printing. This printing technique, mostly referred to in the literature as micro-extrusion [1], has evolved from the conventional fused layer deposition modeling, and consists of the extrusion of a viscous material through a needle, pushed by different mechanisms such as pneumatic pressure, a piston, or a rotating screw [2,3]. Depending on the physico-chemical parameters of the ink, the printing device, and the needle diameter, thread in a wide range of diameters can be used with high precision.

Despite some noteworthy advantages that DIW presents compared with other printing techniques—such as the possibility of printing with biomaterials with high viscosities or its room temperature processing—this technique still presents some limitations. The low printing speed or the need for a specific matching of ink densities to preserve intricate shapes [4], requires the adjustment of specific rheological parameters in order to be suitable for additive manufacturing.

Focusing on the requirements, the designed inks have to be able to decrease their viscosity during extrusion, but also have to recover it quickly to sustain the 3D complex structure, supporting multiple layers without collapsing. In order to determine the suitability of the inks, it is necessary to explore (i) the processability of the ink, also known as “printability”, and (ii) the printing fidelity and the strength of the printed construct to self-sustain a 3D structure post-printing, known as “shape fidelity”. Thus, the evaluation of rheological properties is of utmost importance in DIW technology.

Regarding printability, the desired materials, so as to satisfy the aforementioned complex processing, must be a pseudoplastic fluid with shear-thinning behavior. It must also present viscosity that is low enough during printing in order for the ink to be successfully extruded. Additionally, the yield point, the stress at which the material’s network structure starts to break down and hence starts flowing, also plays a major role in ink printability and controls the successful extrusion of the ink [5,6,7,8,9]. Inks with a very high yield point and high viscosity values could develop a plug-flow regime where the ink slips inside the nozzle as an undeformed plug, blocking the nozzle and thus the printing process itself [10].

Depending on the capacity of the ink to reproduce and maintain the given shape as well as to support multiple layers, a relationship can be established with the complex shear modulus (G*) and more specifically, the storage modulus (G′), the loss modulus (G″), and the loss tangent (tan δ = G″/G′) values derived from spectromechanical analysis. High values of elasticity are correlated with structured systems, which are able to support multiple layers without collapsing due to their solid-like behavior. G′ and tan δ are the two parameters which can be used to pilot the better behavior. In this way, very low tan δ values result in an overly elastic behavior, which can lead to cohesion problems between layers. Additionally, the yield point also has influence on shape fidelity, creating problems for the inks with low values to support the upper layer’s weight, leading to the collapse of the 3D structure by their own weight [5,6].

Thus, this work focuses on understanding the relationship between rheological parameters and printing performance that can help with the design of suitable inks for DIW printing. With this aim, novel waterborne polyurethane–urea (WBPUU) gels with different solid contents based on hydrophobic polycaprolaptone diol (PCL) and hydrophilic poly (ethylene glycol) (PEG) as soft segment, were synthesized and used as printing inks. The water-based nature of this ink avoids the manipulation of volatile organic compound (VOCs), leading to more environmentally friendly process and inks.

The use of WBPUUs with different compositions as printing inks for DIW 3D printing has been reported in the literature in the last years, mostly focused on biomedical applications [9,11] In a recent work, Feng et al. synthesized a biodegradable WBPUU modified with an amino acid, presenting good flexibility and biocompatibility with a potential application in tissue engineering via 3D printing [12]. In another study, Hsieh et al. examined the viability of a thermoresponsive WBPUU that formed a gel near 37 °C without any cross-linker. The ink, with a soft segment composed of poly(D,L-lactide) diol and PCL presented promising results for the embedding of neural stem cells [13].

Additionally, other authors studied the printing process of WBPUU based inks, with the addition of different nanoentities as viscosity modulators, in order to tune the rheological properties and thus the printing performance. In this context, Chen et al. studied two incorporation routes of cellulose nanocrystals (CNC) to a WBPUU so as to obtain an ink suitable for DIW 3D printing. The results showed that the different incorporation routes resulted in different dispositions of the components, leading to inks with different rheological behavior and hence different printing performance [7]. In a previous work, we observed that very low contents of CNC (<1 wt%) added in situ contributed to the shape fidelity by increasing the storage modulus and yield point of the inks, resulting in an improvement in printing performance [14].

Regarding the use of PEG as composition for DIW 3D printing, its use has been mostly reported as a component of inks in biomedical applications. Hung et al. used PEG as a viscosity enhancer to improve the printing process of a WBPUU with a soft segment composed of PCL and polyethylene butylene adipate diol. As a result, a nontoxic and highly elastic scaffold was obtained by 3D printing [9]. Serra et al. studied different ratios of PEG and polylactic acid blends for its processing by 3D printing. The results showed that the incorporation of the PEG has an effect on the properties of the final scaffolds, modifying the mechanical properties as well as increasing biodegradability [15]. Moreover, in a recent work, Ilhan et al. developed a sodium alginate/PEG based ink containing loaded *Satureja cuneifolia*, suitable for the 3D printing of diabetic wound dressing material. In this case, an increase in the PEG content of the ink resulted in a modification of the rheological properties, slightly decreasing ink viscosity as well as the tensile strength, and increasing the strain at the scaffold break [16].

As previously mentioned, despite the recent publication of other works studying WBPUU based inks as 3D printing materials, none of them use the combination of PEG and PCL as the soft segment of the polyurethane in order to promote physical gelation. The incorporation of small amounts of PEG into a PCL based WBPUU allowed for the ink’s physical gelation due to the hydrophilic nature of the PEG, thus producing inks that can be processed by DIW 3D printing.

Additionally, the nature of this kind of water-based systems allowed the incorporation of hydrophilic nanoentities such as cellulose derivatives [17] and/or plant extracts [18,19], which can also be used as natural surfactants for other nanoentities like graphene [20]. This incorporation of nanoentities can lead to the realization of a final printed piece with different properties or even new functionalities. Thus, WBPUU based inks with a PEG:PCL ratio of 0.2:0.8, according to previously optimized results [21], with solid contents ranging from 27 to 46 wt%, were synthesized. The different synthesized inks were extensively characterized from the rheological viewpoint in order to establish a relationship between the structural parameters of the WBPUU based inks, their rheological properties, and their printing performance. Consequently, this would lead to an understanding of the complex behavior of physically crosslinked gel-based inks for DIW and establish valid criteria for designing inks with this novel technology.

## 2. Experimental Part

### 2.1. Materials

Waterborne polyurethane–urea based inks were synthesized using a difunctional polycaprolactone diol (PCL) and a difunctional poly (ethylene glycol) (PEG) (molecular weight from the supplier Mn = 2000 and 1000 g mol^−1^, respectively) as soft segment (SS), both supplied by Sigma-Aldrich (St. Louis, MI, USA). These molecular weights were selected after an optimization process for a better gelation process, where the lower molecular weight PEG showed poor gelling capacity and the high molecular weight PEG resulted in non-homogeneous dispersions. Moreover, a PCL with a molecular weight of 2000 g mol^−1^ was chosen due to its proven excellent performance in WBPPU properties. Portions of 2,2-Bis(hydroxymethyl) propionic acid (DMPA, 98% purity) and ethylene diamine (EDA, 99% purity) used as internal emulsifier and chain extender, respectively (provided by Sigma-Aldrich), along with isophorone diisocyanate (IPDI, 99.5% purity) kindly supplied by Covestro (Leverkusen, Germany), were used as hard segment (HS). Dibutyltin dilaurate (DBTDL) (provided by Sigma-Aldrich), was used as catalyst. The polyols and the DMPA were dried under vacuum at 60 °C for 4 h prior to their use. Triethylamine (TEA, 99.5% purity) was used as a neutralizer of the carboxylic groups of DMPA (provided by Fluka, Buchs, Switzerland), whereas butanone (99.5% purity) (supplied by Panreac, Barcelona, Spain), was used to transfer the neutralized DMPA into the reaction medium and adjust viscosity during the synthesis.

### 2.2. Synthesis of the WBPUU Inks

A WBPUU with a PCL:PEG ratio of 0.8:0.2 was synthesized, following a procedure developed in a previous work [21]. Briefly, the synthesis consisted of a two-step reaction where the former comprised the formation of the prepolymer from PCL, PEG, DMPA, and IPDI, while the latter involved the chain extension with EDA in heterogeneous media once the phase inversion was carried out. The molar ratio of PCL, PEG, DMPA, IPDI, and EDA was 0.8:0.2:1:3.5:1.5. The solid content of the prepared WBPUU was varied from 27 to 46 wt%, adding different amounts of water during the phase inversion. During the chain extension step and with solid contents above 27 wt%, the WBPUU formed a physically crosslinked network. Table 1 summarizes the prepared WBPUU based ink designations and solid content.

### 2.3. DIW 3D Printing of Prepared Inks

For the 3D printing of the prepared systems, a Voladora 3D printing machine (supplied by Tumaker (Gipuzkoa, Spain) and modified to allow the use of extrusion based DIW technology) was used. Samples were printed at a printing speed of 6 mm s^−1^ using a needle with a diameter of 0.8 mm. All samples were loaded into syringes and were centrifuged for 3 min at 3000 rpm prior to printing in order to remove the existing bubbles. The maximum shear rate (${\dot{y}}_{max}$) for a Newtonian fluid on the needle wall can be calculated using Equation (1) [22].

(1) $${\dot{y}}_{max}=\frac{4\;\dot{Q}}{\pi \;r^{3}}\;\;$$

However, in the case of non-Newtonian fluids, the printing process produces a pressure-driven flow that deviates from a parabolic velocity profile. Thus, the shear rate at the wall has to be corrected using the Weissenberg–Rabinowitsch–Mooney equation [23,24]. For a circular capillary profile, the shear rate at the wall can be determined in non-Newtonian fluids by using the following expression:

(2) $${\dot{y}}_{max}=\frac{4\;\dot{Q}}{\pi \;r^{3}}\left(\frac{3n+1}{4n}\right)\;\;$$

where *r* is the needle internal radium; $\dot{Q}$ is the flow rate determined as $\dot{Q}$ = Sr^2^, where S is the printing speed in mm s^−1^; and *n* is the power law exponent. Hence, the shear rate at the walls was determined for the different inks, obtaining a printing shear rate range of 30–50 s^−1^ for all inks, which is a typical value applied during the DIW process [25]. Dog bone shaped samples according to an ISO 527 standard were printed [26]. Additionally, grids of 15 × 15 mm^2^ were also printed to study the integrity of the printing thread.

The validation of the printed pieces was carried out by measuring their dimensions and comparing them with those on the CAD design. In this context, according to criteria described by many authors [27,28,29], an integrity factor was determined by comparing the thickness of the printed piece and the developed model. Additionally, different measurements of the length and width of the CAD model of the dog bone shape were taken as reference in order to analyze the deviations of the printed pieces compared with the CAD model. For that purpose, the dimensions of 3 printed samples per printable system were measured, giving as a result the average of the measurements.

### 2.4. Characterization

The rheological properties of the synthesized inks have been characterized by an R101 Anton Paar rheometer (Anton Paar GmbH, Graz, Austria), which included a temperature chamber and a solvent trap. Rotational rheology was performed by shear rate sweep test (0.01–120 s^−1^) using parallel-plate geometry with a diameter of 50 mm at 22.5 °C. The yield point was determined by a dynamic oscillatory test in a shear stress range sweep (0.09–1500 Pa) at 22.5 °C in a parallel-plate geometry of 25 mm. Moreover, strain sweep tests were performed, fixing the frequency at 1 Hz and varying the strain from 0.01 to 200% in order to determine the linear viscoelastic region. For the frequency sweep, the tests were performed at a fixed strain of 1%, from 0.01 to 10 Hz at 22.5 °C. Finally, structure recovery tests were performed at 22.5 °C to study the recovery capacity of the ink after printing. The test is composed of three stages, where the viscosity is measured based on time at a fixed shear rate, which varies at every stage: (i) 1 s^−1^ for 10 s, (ii) 100 s^−1^ for 10 s, and (iii) 1 s^−1^ for 3 min. The recovery percentage is calculated from the initial viscosity and that measured after the last stage.

## 3. Results and Discussion

**Figure 1:**
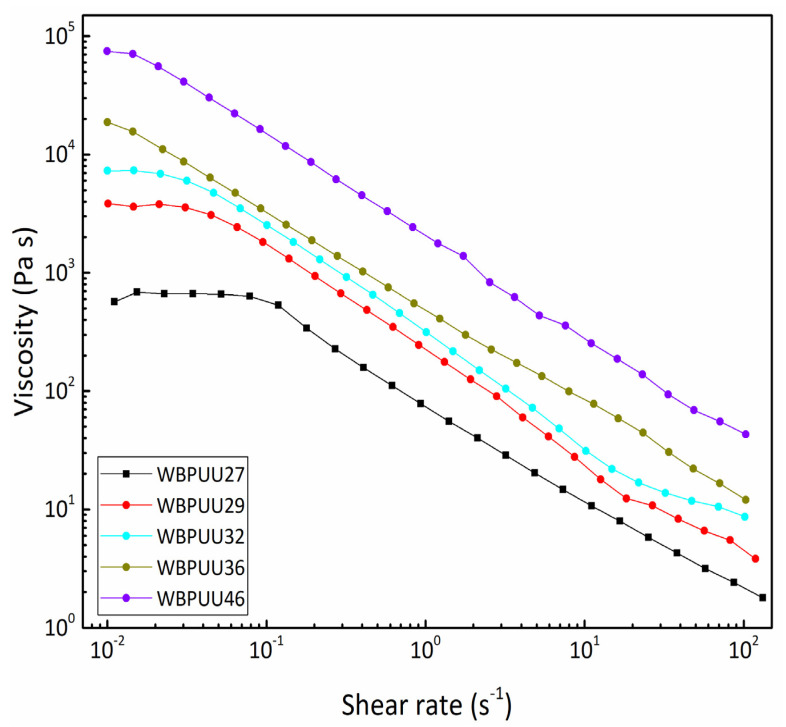
Viscosity as a function of shear rate (T = 22.5 °C) of WBPUUs with different solid contents.

Flow tests were performed for the different prepared WBPUUs (Figure 1). As can be observed, all prepared systems showed shear-thinning behavior in the range of 0.01–120 s^−1^. It is expected that as a result of the increase of the shear rate, the gel network would experience a weakening, resulting in a decrease in viscosity. This behavior was also observed in the literature for other materials [10,30,31] and represents an important property for obtaining printable materials. Comparing systems with different solid contents, it can be concluded that despite all systems presenting the aforementioned shear-thinning behavior, the viscosity measured at low rates (0.01 s^−1^) went up as the solid content of the WBPUU increased. The same behavior was observed in the viscosity measured at the printing shear rate range (30–50 s^−1^). Additionally, WBPUUs with low solid content presented a Newtonian plateau at very low shear rates (between 0.01–0.1 s^−1^ for WBPUU27, 0.01–0.03 s^−1^ for WBPUU29, and 0.01–0.02 s^−1^ for WBPUU32), which was reduced in length and disappeared completely for WBPUU36 and WBPUU42. Gupta et al. observed a similar behavior of the Newtonian plateau with the increase of bentonite clay content in an ethylene–vinyl acetate copolymer/bentonite clay composite [32].

**Figure 2:**
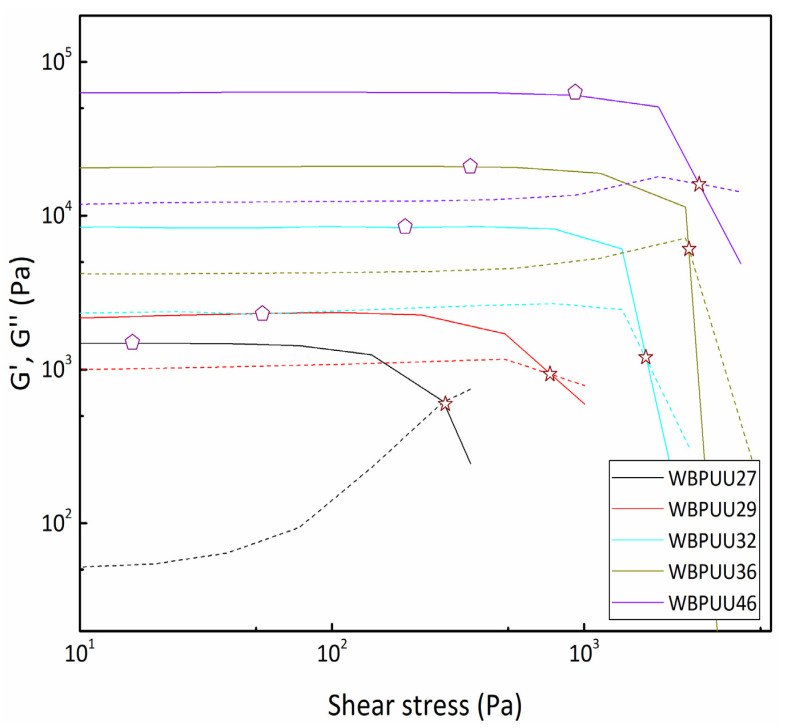
(—) Storage (G′) and (----) loss (G″) moduli as a function of shear stress. Yield and flow point determination of WBPUU inks with different solid contents (T = 22.5 °C). (⬠)Yield point, (☆) Flow point.

In order to determine the yield point of the inks, spectromechanical analysis was performed by a shear stress sweep test (Figure 2). The yield point was determined according to the method reported previously by Cyriac et al., where the yield point is calculated as the stress at which the storage modulus deviates from the linearity [33]. Additionally, the cross-over between G′ and G″ was used to determine the flow point of the inks and to study the behavior of the material during its breakdown. For this, the ratio between the flow point and the yield point, which is known as “flow transition index” (FTI), was calculated [34]. The results, which are displayed in Table 2, presented an increase of both yield and flow point with the increase of the solid content. As the WBPUU particle concentration increased in the dispersion, the interparticle space decreased, leading to a reduction of the interaction volume between the particles, and thus to an increase of the yield point [35,36]. Indeed, the yield point was found to be a power-law function of the volume fraction of solid $\left(\sigma_{y}=k\Phi^{p}\right)$, which was previously observed in the literature [37,38], where $\sigma_{y}$ is the yield point and Φ is associated with the solid content. On the other hand, K and *p* are empirical constants, which in this case are 122.16 and 0.316, respectively.

The increase of the yield point could result in printability problems, taking into account the small diameter of the nozzle, which requires the application of high pressure to extrude the ink successfully. One of the principal issues of not reaching this high pressure is that the ink can behave as a plug form inside the nozzle, resulting in the blocking of the nozzle [10]. In this context, systems above 32 wt% of solid content presented such high yield point values that they could not be extruded successfully through the nozzle without compromising the printing process, as shown below. Additionally, the previously observed higher viscosity values for increasing solid content also presented difficulties in correct extrusion. The use of nozzles with higher inner diameter can solve this issue, allowing for printing systems with higher yield points; however, one of the consequences of this diameter increase is the decrease of the printing resolution, which results in a poorer reproduction of the 3D design.

The yield point also influences the capacity to maintain the shape since very low values can lead to the flow of the lower layers, resulting from the weight of the 3D construction itself. In this case, an increase in the solid content resulted in an increase of the yield point and to a more stable, multilayered 3D construct. Regarding the FTI index, there was a decrease when the solid content was increased, which illustrates the brittle behavior of the soft material as FTI approached 1 [39], leading to the emergence of printing problems.

The obtained data from the flow curves were also adjusted to the Herschel–Bulkley model to determine the consistency index, k, and flow index, *n*, (Figure S1). This model is frequently used to describe yield stress fluids, with $\sigma -\sigma_{y}=k{\dot{y}}^{n}$ where $\sigma_{y}\;$ is the yield point, σ is the shear stress, k corresponds to the consistency index, $\dot{y}$ is the shear rate, and *n* the flow index, with *n* < 1 for shear-thinning fluids [40].

The predicted model gave a good correlation with the experimental curve, obtaining high R^2^ values. The results, which are also displayed in Table 2, showed higher values for k as the solid content increased, suggesting a higher consistency for systems presenting higher solid content. This can provoke problems with the continuous extrusion of the material during 3D printing or result in bad printability [41], as confirmed for systems with solid content above 32 wt%. Regarding *n*, the values under 1 obtained for all systems confirmed the aforementioned shear-thinning behavior observed in the flow curves, which is desirable for 3D printing [42].

Regarding the oscillatory measurements, the linear viscoelastic region (LVR) of the prepared inks was determined by means of a strain sweep test (Figure S2). Upon analysis of the curves, both the storage and the loss moduli were independent of the strain amplitude at low strains, with G′ remaining above G″ in all samples, which confirmed that the material is highly structured [43]. However, the smaller difference between G′ and G″ at LVR for WBPUU27 suggests a weaker structuration for this gel. At strains above 2%, all systems presented a drop in their G′ as a result of the damage in the gel structure, leading to the transition from elastic to viscous behavior when G″ overpassed G′.

**Figure 3:**
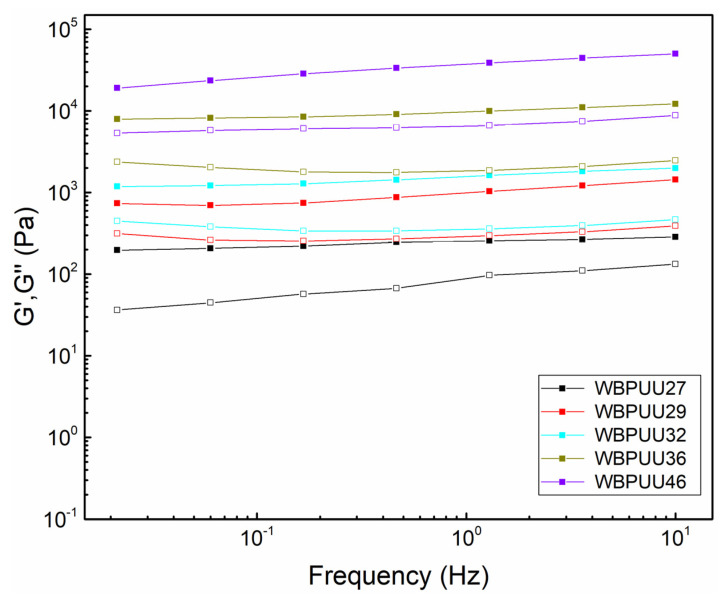
G′ (■) and G″ (□) as a function of frequency (T = 22.5 °C) of WBPUU inks with different solid contents.

Moreover, frequency sweep tests were also performed in the LVR in order to measure both storage and loss moduli of the gels, and thus evaluate the integrity of the inks. The determined G′ and G″ at different frequencies are shown in Figure 3, whereas values measured at 1 Hz of G′, G″, G*, and tan δ are presented in Table 3. The results show that storage, loss, and complex moduli exhibited higher values as the solid content increased, showing a more structured network in agreement with oscillatory strain test results. In all cases, G′ remained over G″ in all frequency ranges, illustrating a predominant elastic behavior [44]. According to the literature, empirically, a storage modulus above 10^3^ Pa is necessary to maintain a stable 3D structure of multiple layers [45]; with the exception of WBPUU27, all the systems presented a storage modulus above this value. Additionally, according to Álvarez-Castillo et al., inks presenting a complex modulus below 10^3^ Pa were too weak to keep the shape, whereas values over 10^5^ Pa were too stiff to be properly extruded [46]. In this case, the WBPUU27 was once again the only system to present values under this range.

Regarding tan δ, all systems except for WBPUU27 presented values in the range between 0.1 and 0.3, showing more elastic behavior compared with WBPUU27. This system, which presented a tan δ of 0.52 at 1 Hz, exhibited more viscous behavior that led to problems in shape retention since the material flowed after printing, as shown below. A small increase in the solid content boosted physical gelation, increasing the difference among storage and loss moduli values. Consequently, the tan δ experienced a decrease as the solid content increased, thus converting viscous behavior to a predominantly elastic one, which resulted in an ink more capable of maintaining its shape.

**Figure 4:**
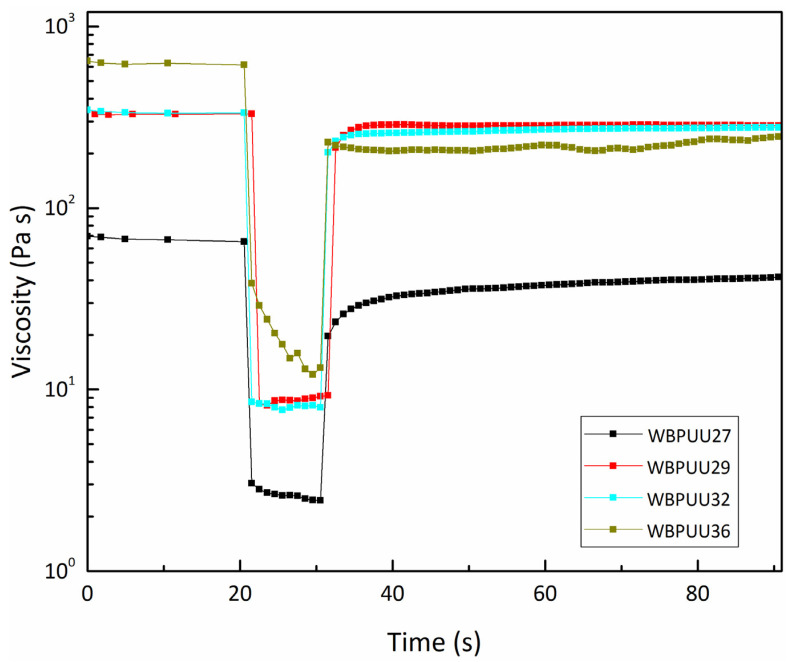
Structure recovery test of WBPUU based inks with different solid contents (T = 22.5 °C).

The structural integrity and shape fidelity of the printable WBPUUs were tested by determining their recovery capacity in order to predict their performance during printing. According to the obtained results (seen in Figure 4) and recovery values (gathered in Table 3), the WBPUU29 and WBPUU32 showed fast recovery values of 84% and 83%, respectively, showing an increase compared with WBPUU27, which showed a slow time-dependent recovery of 63%. Peak et al. reported that an initial viscosity recovery of 80% is significant for 3D printing [47], confirming the viability of these inks for DIW. However, WBPUU36 experienced a decrease in recovery, obtaining a value of 40%. This decrease happened due to a material slip during the high shear rate period, which was noticeable since a decrease in viscosity was observed at the stage of 100 s^−1^ shear rate.

**Figure 5:**
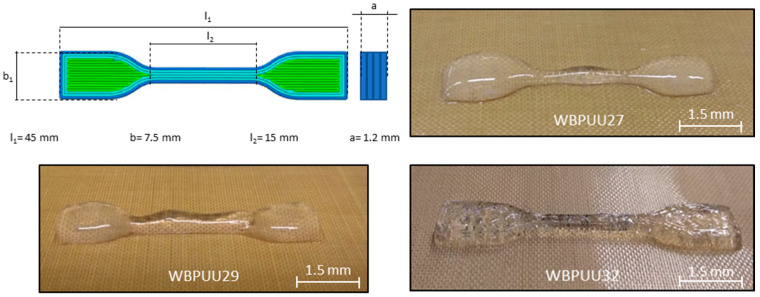
CAD model and dimensions, and printed pieces of WBPUU inks with different solid contents.

The printing performance of the developed inks was evaluated by extrusion based DIW. Systems presenting yield points of 195 Pa or below this value were printed successfully, forming a continuous and homogeneous thread when the ink was extruded, whereas those with higher yield point values could not be printed through the nozzle (WBPUU36 and WBPUU46). The digital images of the printed pieces are displayed in Figure 5. Regarding shape fidelity, from among the printable systems, WBPUU32 presented the best results. Studying the dimensions of the printed pieces (displayed in Table 4), the piece printed with the WBPUU32 system had a higher correlation with the CAD design in all axes, presenting a high integrity factor as well as similar dimensions compared with the model. However, other systems presented a poorer correlation, obtaining a lower integrity factor as the solid content decreased, and presenting higher values for the length and width due to the spreading of the inks. The higher yield point and storage modulus observed in WBPUU32 allowed for better support of the weight of the upper layers, leading to better shape fidelity and preventing the spreading of the ink, as observed in inks with lower solid content. The high structure recovery value observed for this ink after applying stress, as well as its adequate yield point value confirmed the building of a strong structure after the extrusion process. This resulted in a quick recovery of its initial state, and thus to the realization of a 3D construct that can support multiple layers and successfully maintain the shape designed by the software.

**Figure 6:**
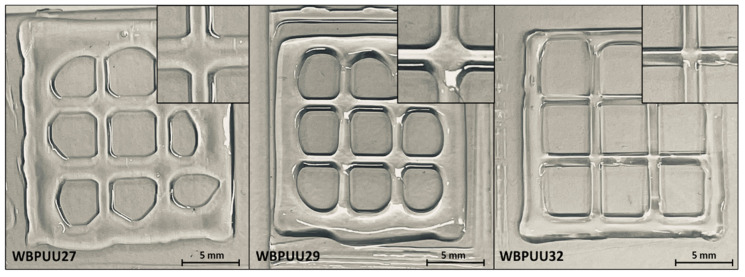
Digital images of printed grids with different WBPUU inks. Magnification: detail of the thread crossings.

Additionally, and in order to demonstrate the integrity of the formed thread by the different prepared inks, grids were printed for WBPUU27, WBPUU29, and WBPUU32. The digital images taken just after printing the grids (shown in Figure 6) confirm the higher structuration of WBPUU32 as compared to systems presenting lower solid content. In the case of WBPUU29 and WBPUU27, the ink collapsed after being printed and flowed, resulting in the spread of the thread, which can be observed clearly at the intersections. In contrast, the grid printed with WBPUU32 showed good shape fidelity, presenting the thread with a homogenous appearance and maintaining its shape after being printed without collapsing. In this case, both threads can be clearly recognized, maintaining the printed pattern of the ink at the intersection.

## 4. Conclusions

In this work, inks based on a novel waterborne polyurethane urea combining in the soft segment of hydrophilic PEG and hydrophobic PCL were synthesized. The solid content of the ink was varied during the synthesis in order to explore it as a design variable. A thorough rheological study was carried out to establish the optimal parameters for an extrusion-based DIW printing technology. The analyzed inks were subsequently printed in order to study their capacity to reproduce complex 3D constructs with good shape fidelity, and thus establish a relationship between the rheological behavior and the printing performance.

All the studied inks confirmed their suitability for DIW printing, presenting a shear-thinning behavior. The increase of the solid content of the ink resulted in an increase of both yield point and viscosity, which resulted in printability limitations for inks with high solid contents. The storage modulus increased and the tan δ decreased as the solid content of the systems increased, leading to more structured and solid-like inks, which can better maintain the shape and support multiple layers. In this case, the WBPUU32 showed the best printing performance, combining good printability and an excellent shape fidelity that has led to the realization of an accurate multilayered reproduction of the 3D printing design. Additionally, rheology proved to be a useful tool for designing suitable inks for DIW printing, allowing for the creation of a criterion of rheological parameters that can be related to printing performance.

## Figures and Tables

**Table 1:** Designation, and calculated solid content of prepared WBPUU based inks.

Ink	Calculated Solid Content (wt%)
WBPUU27	27
WBPUU29	29
WBPUU32	32
WBPUU36	36
WBPUU46	46

**Table 2:** Yield point, flow point, flow transition index, and parameters obtained from the adjustment of WBPUU inks with increasing solid content to the Herschel–Bulkley model ($\sigma -\sigma_{y}=k{\dot{y}}^{n})$.

System	Yield Point (Pa)	Flow Point (Pa)	FTI	Herschel–Bulkley Model		
				K Index	N Index	R^2^
WBPUU27	17	282	16	72	0.24	0.97
WBPUU29	44	562	13	177	0.15	0.92
WBPUU32	195	1755	9	238	0.14	0.84
WBPUU36	354	2608	7	303	0.37	0.98
WBPUU46	921	2857	3	1032	0.20	0.94

**Table 3:** Storage modulus, loss modulus, complex modulus, and tan δ values determined at 1 Hz, and structure recovery percentage of inks with different solid contents.

System	Storage Modulus (Pa)	Loss Modulus (Pa)	Complex Modulus (Pa)	Tan δ	Structure Recovery (%)
WBPUU27	206	106	231	0.52	63
WBPUU29	1033	296	1075	0.29	84
WBPUU32	1618	359	1657	0.22	83
WBPUU36	9966	1865	10,139	0.19	40
WBPUU46	38,738	6630	39,301	0.17	-

**Table 4:** Comparison of the dimension of the printed pieces with the CAD model.

System	Dimensions of Printed Pieces (mm)				Integrity Factor
	l_1_	l_2_	b_1_	a	
CAD model	45	15	7.50	1.2	-
WBPUU27	45.10 ± 0.03	15.01 ± 0.01	7.55 ± 0.02	0.93 ± 0.02	0.78 ± 0.05
WBPUU29	45.03 ± 0.01	15.01 ± 0.01	7.53 ± 0.01	1.02 ± 0.03	0.85 ± 0.02
WBPUU32	44.99 ± 0.01	15.00 ± 0.00	7.50 ± 0.01	1.19 ± 0.01	0.98 ± 0.01

## Data Availability

The data presented in this study are available on request from the corresponding author.

## Supplementary Materials

The following are available online at https://www.mdpi.com/article/10.3390/ma14123287/s1. Figure S1: Adjustment to the Herschel-Bulkley model of WBPUU29 flow curve, Figure S2: G′ (■) and G″ (□) as a function of strain (T = 22.5 °C) of WBPUU inks with different solid content at 1Hz.
