# Supplementary material for: Design of a Waterborne Polyurethane–Urea Ink for Direct Ink Writing 3D Printing
Source: Materials (Basel). 2021 Jun 14;14(12):3287. doi: 10.3390/ma14123287 (PMC8232083; doi:10.3390/ma14123287)
Supplement: Supplementary file 1 [file materials-14-03287-s001.zip › materials-1250991-supplementary.pdf]

Supplementary Materials

Design of a Waterborne Polyurethane–Urea Ink for Direct Ink Writing 3D Printing

Julen Vadillo [1,2], Izaskun Larraza [1], Tamara Calvo-Correas [1], Nagore Gabilondo [1], Christophe Derail [2,*] and Arantxa Eceiza [1,*]

1 Materials + Technologies Research Group (GMT), Department of Chemical and Environmental Engineering, Faculty of Engineering of Gipuzkoa, University of Basque Country, Plz. Europa 1, 20018 Donostia-San Sebastian, Spain; julen.vadillo@univ-pau.fr (J.V.); izaskun.larraza@ehu.eus (I.L.); tamara.calvo@ehu.eus (T.C.-C.); nagore.gabilondo@ehu.eus (N.G.)

2 E2S UPPA, IPREM, UMR5254, Centre National de la Recherche Scientifique (CNRS), Institut des Sciences Analytiques & de PhysicoChimie pour l'Environnement & les Matériaux, Universite de Pau et Pays de l'Adour, 64000 Pau, France

* Correspondence: christophe.derail@univ-pau.fr (C.D.); arantxa.eceiza@ehu.eus (A.E.)

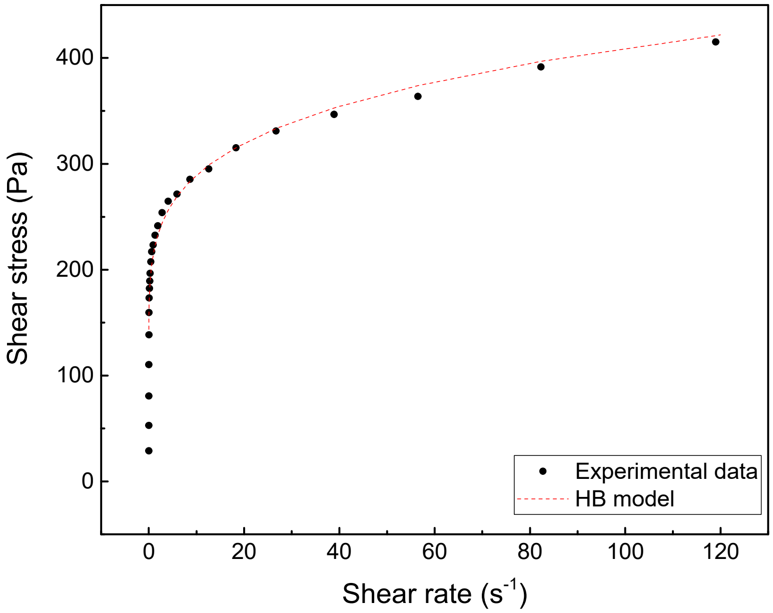

Figure S1. Adjustment to the Herschel-Bulkley model of WBPUU29 flow curve.

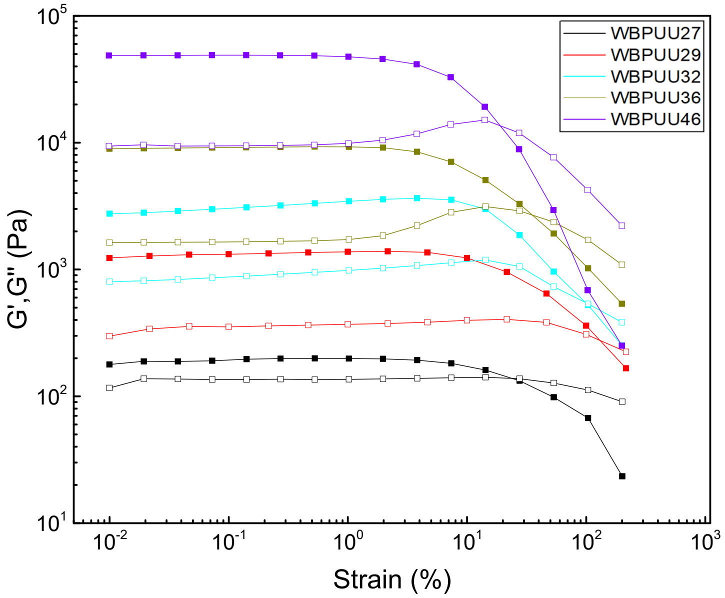

Figure S2. G′ (■) and G′′ (□) as a function of strain (T = 22.5 °C) of WBPUU inks with different solid content at 1Hz.
